# Resolution of Recurrent Severe Hypoglycemia After Surgical Excision of a Primary Hepatic Neoplasm of Uncertain Histogenesis in a Dog: A Case Report

**DOI:** 10.3390/vetsci13080822

**Published:** 2026-08-18

**Authors:** Hyeong-Wook Moon, Chi-Youn Song, Jong-Myung Lee, Kwang-Rae Jo, Geun-Bo Park, Hye-Soo Shim, Hwi-Yool Kim, Jung-Moon Kim

**Affiliations:** 1 Department of Veterinary Surgery, College of Veterinary Medicine, Konkuk University, Seoul 05029, Republic of Korea; 2 Suwon Barun Animal Medical Center, Suwon 16391, Republic of Korea

**Keywords:** dog, primary hepatic neoplasm, tumor-associated hypoglycemia, suspected non-islet-cell tumor hypoglycemia, surgical excision, immunohistochemistry

## Abstract

Primary hepatic neoplasms in dogs, while uncommon, are occasionally associated with severe hypoglycemia, although the underlying mechanism may be difficult to establish. A 7-year-old castrated male Maltese dog weighing 3.6 kg was referred for recurrent bilateral pelvic limb weakness, episodic collapse, and abdominal distension. Severe hypoglycemia was documented, with a blood glucose concentration of 26 mg/dL. Computed tomography revealed a large mass arising from the right lateral liver lobe, with no identifiable pancreatic mass or distant metastasis. The serum insulin concentration measured during hypoglycemia was low at 1.0 μU/mL, making insulinoma less likely. Glycemic control temporarily or partially improved during prednisolone treatment; however, severe hypoglycemia recurred after prednisolone discontinuation before surgery. The hepatic mass was surgically excised. Dextrose-containing intravenous fluids were maintained for 6 h postoperatively, and no recurrent clinically significant hypoglycemia was documented after their discontinuation. Histopathologic examination revealed a poorly differentiated neoplasm with mesenchymal morphology, occasional cytoplasmic vacuolation, and no unequivocal lipoblasts. The neoplastic cells showed diffuse vimentin immunoreactivity, weak cytoplasmic S-100 immunoreactivity in a subset of cells, and no immunoreactivity for smooth-muscle actin or hepatocyte paraffin 1. These findings raised the possibility of adipocytic differentiation but were insufficient to establish a specific tumor lineage; therefore, the lesion was classified as a primary hepatic neoplasm of uncertain histogenesis. At 247 days after surgery, the dog remained normoglycemic without supplemental glucose or glucocorticoid treatment, and no imaging evidence of local recurrence or distant metastasis was identified. The postoperative course supported an association between the hepatic neoplasm and hypoinsulinemic hypoglycemia. Non-islet-cell tumor hypoglycemia was suspected but remained unconfirmed because an insulin-like growth factor-mediated mechanism was not demonstrated.

## Simple Summary

Primary hepatic neoplasms are occasionally associated with severe hypoglycemia in dogs, although the underlying mechanism may be difficult to establish. This report describes a seven-year-old Maltese dog that repeatedly developed dangerously low blood sugar, resulting in weakness of both pelvic limbs, abrupt sitting, and collapse. Diagnostic imaging revealed a large liver mass. Blood testing showed a low insulin concentration during hypoglycemia, making an insulin-secreting pancreatic tumor less likely. The mass was surgically removed. Histopathology and immunohistochemistry supported classification of the mass as a primary hepatic neoplasm, although the exact tumor type could not be conclusively determined. After postoperative glucose-containing fluids were discontinued, clinically significant hypoglycemia did not recur, and the dog remained clinically well without further episodes of weakness or collapse during the 247-day follow-up period. No imaging evidence of tumor recurrence or spread to other organs was identified at 247 days. This case suggests that a primary hepatic neoplasm should be considered as a possible contributor to recurrent severe hypoglycemia when a liver mass is identified and other common causes are not supported. Moreover, it suggests that surgical excision may have both diagnostic and therapeutic value.

## 1. Introduction

Hypoglycemia is a clinically important metabolic abnormality in dogs and may manifest as weakness, tremors, collapse, seizures, altered mentation, or other neurologic signs [1,2]. Potential causes and mechanisms of hypoglycemia include excessive insulin activity, impaired hepatic glucose production, increased peripheral glucose utilization, sepsis, endocrine disorders, and neoplasia [3,4,5,6]. Among neoplastic causes, insulinoma is the most widely recognized cause of severe hypoglycemia in dogs and is typically supported by an inappropriately normal or increased insulin concentration during hypoglycemia [3,4].

However, hypoglycemia has also been reported in dogs with several nonpancreatic neoplasms, including hepatic tumors, smooth-muscle tumors, mammary carcinoma, renal tumors, vascular tumors, and melanoma [1,7,8,9]. Among hepatic neoplasms, hypoglycemia has been reported in dogs with hepatocellular carcinoma, hepatocellular adenoma, hepatic leiomyosarcoma, and hepatic hemangiosarcoma [1,10,11]. Postoperative resolution of hypoglycemia or normalization of blood glucose concentrations has been documented in selected dogs following surgical resection of hepatocellular neoplasms, including hepatocellular carcinoma and hepatocellular adenoma [1,11,12,13,14]. These reports indicate that hepatic neoplasia should be considered a potential contributor to recurrent hypoglycemia when a hepatic mass is identified, and other common causes are not supported.

Definitive classification of poorly differentiated primary hepatic neoplasms may be challenging, particularly when morphologic and immunophenotypic findings do not conclusively support a specific tumor lineage [15,16,17]. Against this background, this report describes the diagnostic evaluation, surgical management, histopathologic and immunohistochemical assessment, and postoperative clinical course of a dog with recurrent severe hypoglycemia and a large primary hepatic neoplasm of uncertain histogenesis.

## 2. Case Description

A 7-year-old castrated male Maltese dog weighing 3.6 kg was referred for evaluation of progressive bilateral pelvic limb weakness and abdominal distension. Pelvic limb weakness had developed approximately 1 week before presentation. A similar episode had occurred approximately 1 year earlier and had reportedly improved after corticosteroid treatment at another hospital; however, the specific corticosteroid, dose, and treatment duration could not be confirmed. On the day of presentation, the pelvic limb weakness worsened, prompting evaluation at a local veterinary clinic before referral to our hospital for further assessment. According to the owner, the dog showed episodes of collapsing or abruptly sitting because of weakness in both pelvic limbs, but no obvious lameness was observed. No history of trauma or other relevant medical conditions was reported. Although the dog had recently received treatment for dermatologic disease, the medication name, dose, and exact timing of administration could not be confirmed. No medications were being administered at the time of presentation. The only gastrointestinal sign was a single episode of vomiting. The owner stated that the most recent blood examination had been performed approximately 1 year earlier, and no remarkable abnormalities had been identified at that time.

On presentation, the dog was mildly depressed and had difficulty rising, with a tendency to collapse into sternal recumbency because of hindlimb weakness. Cardiac auscultation revealed no murmur. The capillary refill time was within normal limits, and systolic blood pressure was approximately 120 mmHg. However, the heart rate was 60 beats/min, consistent with bradycardia. Following hospitalization and initial stabilization, the heart rate increased to 84–108 beats/min.

Blood testing was performed, including complete blood count (CBC), serum biochemistry, blood gas and electrolyte analysis, C-reactive protein (CRP) concentration, canine pancreas-specific lipase (cPL), D-dimer concentration, total thyroxine (T4) concentration, free thyroxine concentration, and adrenocorticotropic hormone (ACTH) stimulation testing. Complete blood counts were performed using a ProCyte Dx Hematology Analyzer (IDEXX Laboratories, Inc., Westbrook, ME, USA). Serum biochemical analyses were performed using a FUJI DRI-CHEM NX700V analyzer (FUJIFILM Corporation, Tokyo, Japan), and blood gas and electrolyte analyses were performed using an ABL9 blood gas analyzer (Radiometer Medical ApS, Brønshøj, Denmark). Serum CRP and D-dimer concentrations were measured using a Vet Chroma immunoassay analyzer (Anivet Diagnostics Inc., Chuncheon, Republic of Korea), whereas total T4 and cortisol concentrations were measured using a FUJI DRI-CHEM IMMUNO AU10V analyzer (FUJIFILM Corporation, Tokyo, Japan). Canine pancreas-specific lipase was assessed using a SNAP cPL test (IDEXX Laboratories, Inc., Westbrook, ME, USA). The blood glucose concentration was found to be markedly decreased at 26 mg/dL (reference interval [RI]: 75–128 mg/dL). Mild anemia (hematocrit: 32.1%; RI: 37.3–61.7%), thrombocytopenia (70 × 10^3^/μL; RI: 148–484 × 10^3^/μL), leukocytosis (18.07 × 10^3^/μL; RI: 5.05–16.76 × 10^3^/μL) with neutrophilia, and an increased C-reactive protein concentration (19.84 mg/L; RI: 0–10 mg/L) were also observed. The serum alanine aminotransferase (ALT), aspartate aminotransferase (AST), alkaline phosphatase (ALP), and gamma-glutamyl transferase (GGT) activities were 39, 41, 129, and 2 U/L, respectively, and the total bilirubin concentration was 0.3 mg/dL. The serum albumin concentration was 3.2 g/dL (RI: 2.6–4.0 g/dL), whereas the blood urea nitrogen concentration was decreased at 3.8 mg/dL (RI: 9.2–29.2 mg/dL). These findings were not consistent with marked hepatocellular injury, cholestasis, or advanced hepatic failure at presentation. The SNAP cPL result was normal, and the D-dimer concentration was within the reference interval at 219 ng/mL (RI: 0–250 ng/mL). The total T4 concentration was decreased at 0.73 μg/dL (RI: 1.3–2.9 μg/dL), whereas the free T4 concentration measured at KVL (Seoul, Republic of Korea) was within the laboratory reference interval. This pattern was considered compatible with nonthyroidal illness rather than primary hypothyroidism. An ACTH stimulation test was performed using tetracosactide (Synacthen; Dalim BioTech, Seoul, Republic of Korea) administered intravenously at 5 μg/kg. Serum cortisol concentrations were measured immediately before ACTH administration and 1 h after administration. The pre- and post-ACTH cortisol concentrations were 9.5 μg/dL and 20.8 μg/dL, respectively. An assay-specific in-house reference interval for post-ACTH cortisol concentration had not been established at our hospital; therefore, the result was interpreted using a published diagnostic cutoff for canine hypoadrenocorticism. The post-ACTH cortisol concentration was well above the diagnostic cutoff of 2 μg/dL, making hypoadrenocorticism unlikely [18]. Because hypoglycemia was the predominant clinical abnormality, a serum sample was collected immediately after presentation during documented hypoglycemia and before the administration of dextrose, prednisolone, or any other treatment, and was submitted to GREEN VET (Yongin, Republic of Korea) for insulin measurement. The separated serum sample was stored frozen and transported frozen to the referral laboratory. The test was listed by the laboratory as “Insulin (Canine)” and was performed using a human-use immunoradiometric assay applied to canine serum. The serum insulin concentration was 1.0 μU/mL. The laboratory confirmed that this represented a measured value rather than a value assigned at the lower reporting limit. The laboratory report listed a fasting canine reference interval of 8.1–32.0 μU/mL; therefore, the measured concentration was below the laboratory-provided reference interval. Approximately 7 days elapsed from sample collection to result reporting. Because this was an outsourced referral test, the instrument model, reagent manufacturer, formal canine analytical validation data, analytical sensitivity, exact storage temperature, and exact interval from sample collection to analytical processing could not be obtained from the laboratory despite further inquiry.

**Figure 1:**
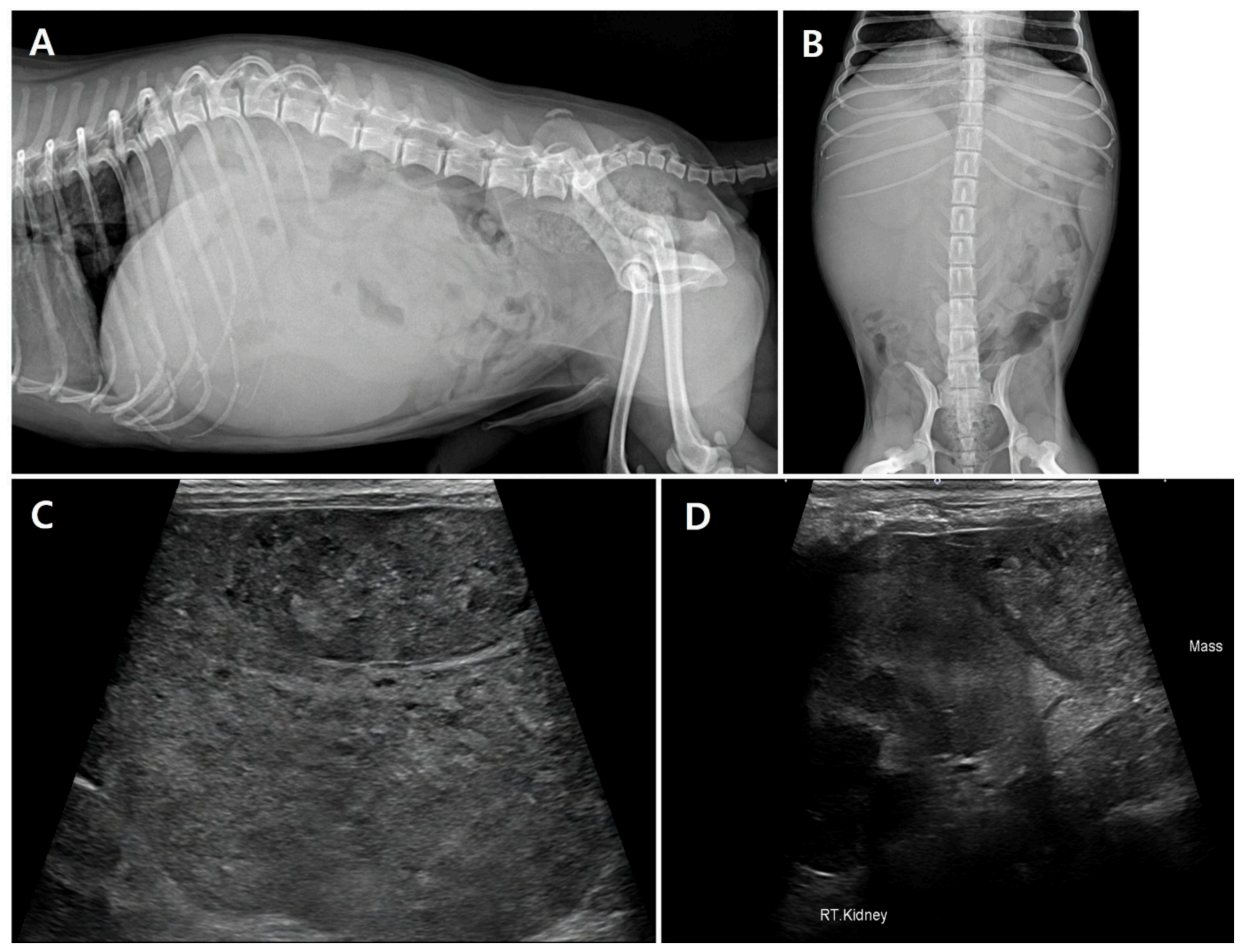
Abdominal radiographic and ultrasonographic findings. (**A**) Right lateral and (**B**) ventrodorsal abdominal radiographs showing a large soft-tissue opaque mass occupying the right cranial abdomen and causing marked displacement of adjacent abdominal viscera. (**C**) Longitudinal ultrasonographic image showing a large heterogeneous hepatic mass with mixed echogenicity. (**D**) Transverse ultrasonographic image showing dorsal displacement of the right kidney by the adjacent hepatic mass.

Thoracic and abdominal radiographs were obtained. Abdominal radiographs demonstrated a large soft-tissue opaque mass centered in the right cranial abdomen, causing marked displacement of adjacent abdominal viscera (Figure 1A,B). Abdominal ultrasonography revealed a large heterogeneous mass, measuring more than 10 × 5 × 4 cm, arising from the right hepatic division (Figure 1C,D). Because of the large size of the mass, its precise lobe of origin could not be determined ultrasonographically.

In addition, a large amount of immobile biliary sludge and multiple choleliths were observed within the gallbladder. Both kidneys showed increased cortical echogenicity with reduced corticomedullary distinction. Because concurrent renal biochemical variables were within their respective reference intervals, these renal ultrasonographic changes were considered nonspecific and unlikely to account for the presenting clinical signs. The pancreas appeared diffusely thickened (17.4 mm) and hypoechoic, with increased echogenicity of the surrounding mesenteric fat.

For further characterization of the hepatic mass, assessment of potential metastatic disease, and surgical planning, contrast-enhanced computed tomography (CT) was performed on the following day using a 16-slice CT scanner (Aquilion Start, model TSX-037A; Canon Medical Systems Corporation, Otawara, Japan). The dog was positioned in sternal recumbency, and images were acquired using 120 kV, 150 mA, a slice thickness of 1.0 mm, and a reconstruction interval of 1.0 mm. After acquisition of precontrast images, iohexol (Omnipaque 300, 300 mg I/mL; GE Healthcare AS, Oslo, Norway) was administered through a cephalic vein at a dose of 3 mL/kg and an injection rate of 2.0 mL/s using a power injector. Bolus tracking was performed using a region of interest placed within the descending aorta. The arterial acquisition was triggered when aortic attenuation reached 150 HU, and the portal venous and delayed phases were acquired 40 and 130 s after initiation of the arterial acquisition, respectively.

**Figure 2:**
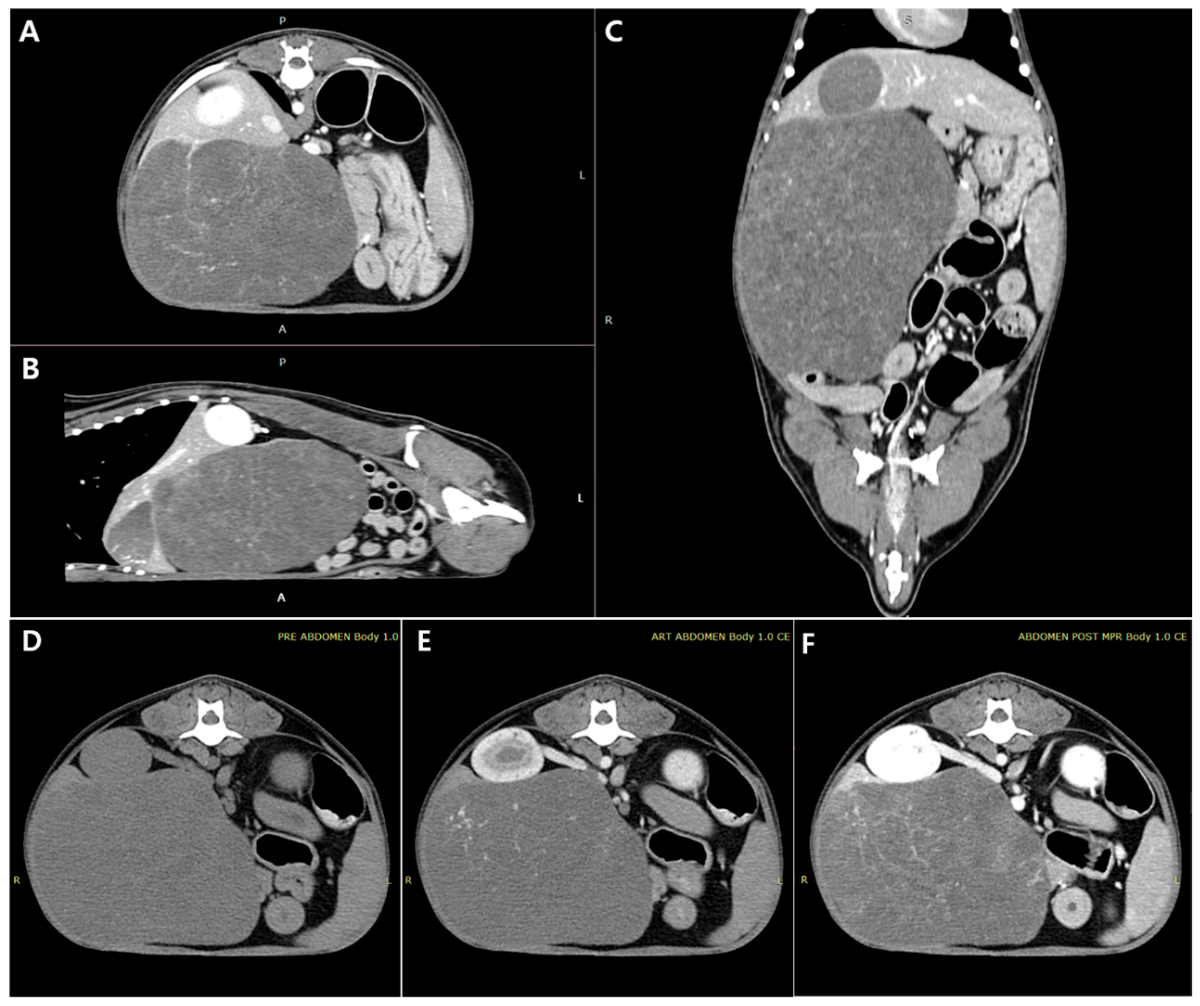
Computed tomography (CT) findings of the hepatic mass. (**A**) Transverse, (**B**) sagittal, and (**C**) dorsal reconstructed contrast-enhanced CT images showing a large heterogeneous hypoenhancing mass arising from the right lateral liver lobe with marked displacement of abdominal viscera. (**D**–**F**) Selected transverse CT images obtained before contrast administration (**D**), during the arterial phase (**E**), and during the delayed phase (**F**). The mean attenuation values of the mass in the displayed phases were approximately 28, 23, and 48 Hounsfield units (HU), respectively. The mass remained markedly hypoattenuating relative to the normal hepatic parenchyma on the postcontrast images, and no areas of macroscopic fat attenuation were identified within the mass.

CT localized the mass to the right lateral liver lobe and revealed a large heterogeneous lesion measuring 11.3 × 8.9 × 6.7 cm (Figure 2A–C). The mean precontrast attenuation value of the mass was approximately 28 HU. In the arterial, portal venous, and delayed phases, the mean attenuation values of the mass were approximately 23, 32, and 48 HU, respectively, compared with approximately 63, 87, and 146 HU in the normal hepatic parenchyma during the corresponding phases. Therefore, the mass remained markedly hypoattenuating relative to the normal hepatic parenchyma throughout all postcontrast phases. No areas of macroscopic fat attenuation were identified within the mass. Most abdominal viscera were displaced leftward because of the marked mass effect. Multiple choleliths were present within the gallbladder and cystic duct, with preserved tapering of the biliary tract. Alongside the absence of hyperbilirubinemia or marked hepatobiliary enzyme abnormalities, these findings indicated that the biliary sludge and choleliths were nonobstructive at the time of imaging. No pancreatic mass or other significant pancreatic abnormality was identified on CT. The apparent pancreatic enlargement and abnormal echogenicity observed ultrasonographically were considered equivocal, as the marked visceral displacement and altered anatomic relationships caused by the large hepatic mass may have resulted in partial superimposition of the pancreatic limbs and overestimation of pancreatic thickness. Enlargement of the hepatic lymph node was also noted. The lymph node was not sampled, and the specific reason for non-sampling was not documented in the contemporaneous operative record. Because reactive and metastatic lymph-node enlargement could not be distinguished by imaging alone, the regional lymph-node status was considered indeterminate. No pulmonary or other distant metastatic lesions were identified on the available imaging studies.

The dog was hospitalized for management of recurrent hypoglycemia. The blood glucose concentration was monitored every 1–2 h. When the blood glucose concentration decreased below 60 mg/dL, supplemental glucose was administered as either a 20% glucose solution (1 mL/kg, slowly intravenously) or a 50% glucose solution (1 mL/kg, orally). Intravenous fluid therapy containing 5% dextrose in an isotonic crystalloid solution was administered at 2.5–3.75 mL/kg/h, and prednisolone was administered orally at 0.5 mg/kg every 12 h. Approximately 10 hypoglycemic episodes requiring additional glucose supplementation were documented during the first day of hospitalization, with a nadir blood glucose concentration of 22 mg/dL. The frequency of the episodes gradually decreased during hospitalization, although approximately four additional glucose administrations were still required on the fourth day. Amoxicillin–clavulanate (12.5 mg/kg every 12 h) was administered empirically because a concurrent inflammatory or infectious process could not initially be excluded. Esomeprazole (0.5 mg/kg every 12 h) was administered for acid suppression because of the recent vomiting episode and concurrent glucocorticoid treatment. Surgery was not performed immediately because the serum insulin concentration from the hypoglycemic sample was still pending, and insulinoma remained an important differential diagnosis. On the fourth day of hospitalization, the dog was discharged at the owner’s request. Before discharge, the owner was informed of the risk of recurrent severe hypoglycemia. The dog’s usual total daily food ration was divided into six meals. The owner was instructed to obtain a home blood glucose meter and to measure the blood glucose concentration immediately if signs suggestive of hypoglycemia, such as lethargy, weakness, abrupt sitting, collapse, tremors, altered mentation, or seizures, occurred. If hypoglycemia was confirmed, a 20% sucrose solution was to be administered orally as an emergency measure. Immediate veterinary evaluation was recommended if hypoglycemia persisted despite oral sucrose administration, if the clinical signs failed to improve or worsened, or if neurologic signs developed. Re-examination was recommended within 2–3 days after discharge. However, the dog was presented 10 days later because the owner had observed no additional clinical abnormalities during the intervening period. At discharge, prednisolone (0.5 mg/kg every 12 h), amoxicillin–clavulanate (12.5 mg/kg every 12 h), and esomeprazole (0.5 mg/kg every 12 h) were prescribed.

Subsequently, the serum insulin concentration measured in the sample obtained during the hypoglycemic episode was confirmed to be low (1.0 μU/mL). At the follow-up evaluation, while prednisolone treatment was continued, the blood glucose concentration was 102 mg/dL, and the D-dimer concentration was 348.45 ng/mL. The serum ALT, AST, ALP, and GGT activities were 41, 30, 233, and 14 U/L, respectively. The low serum insulin concentration measured during hypoglycemia made insulinoma less likely, and the presence of the large hepatic mass raised concern for tumor-associated hypoinsulinemic hypoglycemia. Therefore, surgical resection was planned.

The dog was re-presented to the hospital the day before surgery for preoperative reassessment and stabilization; its blood glucose concentration was 89 mg/dL. The CBC findings were unremarkable, and the previous thrombocytopenia had resolved. The CRP and D-dimer concentrations were within reference intervals. The serum ALT, AST, ALP, and GGT activities were 188, 29, 302, and 21 U/L, respectively. After admission, prednisolone was discontinued before surgery because of concerns regarding potential perioperative glucocorticoid-associated adverse effects, particularly impaired wound healing and increased susceptibility to infection. Following glucocorticoid withdrawal, hypoglycemia recurred, with blood glucose concentrations ranging from 22 to 49 mg/dL. Therefore, intravenous fluid therapy containing 5% dextrose and additional glucose supplementation was resumed, and surgery was performed on the following day.

Preoperative coagulation testing was performed using a QuickVet Specialty Analyzer with QuickVet COAG PT/aPTT cartridges (Zoetis Denmark ApS, Farum, Denmark). The prothrombin time was 17.1 s (RI: 14–19 s), and the activated partial thromboplastin time was 99.5 s (RI: 75–105 s); both values were within their respective reference intervals. Preoxygenation was not performed before anesthetic induction. Midazolam (0.2 mg/kg, IV) was administered as premedication, and anesthesia was induced with alfaxalone (2 mg/kg, IV). Additional perioperative medications included amoxicillin–clavulanate (12.5 mg/kg, IV) and famotidine (0.5 mg/kg, IV). After endotracheal intubation, anesthesia was maintained with isoflurane in oxygen under spontaneous ventilation. Heart rate, respiratory rate, oxygen saturation, end-tidal carbon dioxide concentration, noninvasive blood pressure, and body temperature were continuously monitored and remained clinically acceptable throughout anesthesia. A 5% dextrose-containing isotonic crystalloid solution was administered at 5 mL/kg/h. Because the blood glucose concentration immediately before anesthesia was 39 mg/dL, a 20% glucose solution was administered intravenously at 1 mL/kg before surgery. The blood glucose concentration was measured once intraoperatively and was 90 mg/dL. Perioperative analgesia consisted of tramadol (4 mg/kg, IV), and no additional analgesic was administered intraoperatively.

**Figure 3:**
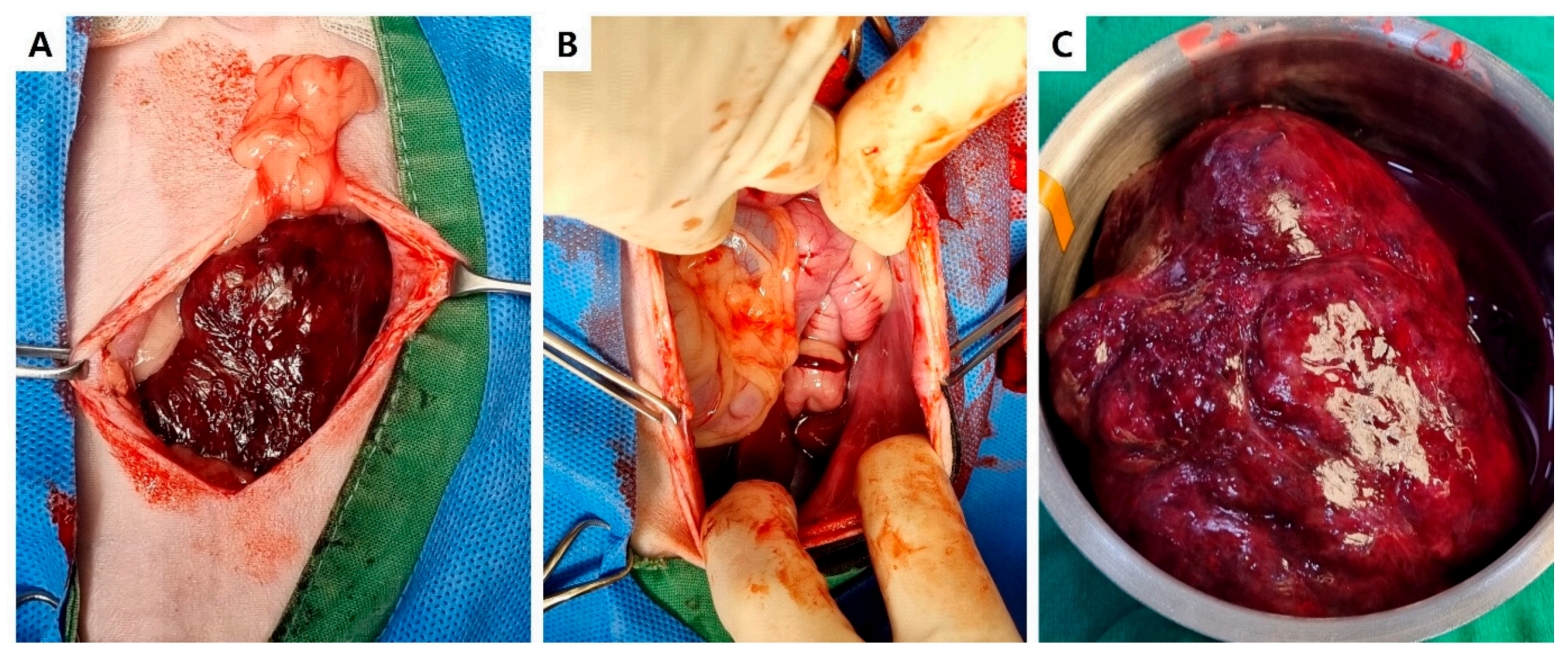
Intraoperative and gross appearance of the hepatic mass. (**A**) Intraoperative appearance of the large hepatic mass occupying most of the surgical field immediately after ventral midline celiotomy. (**B**) Abdominal cavity after removal of the mass. (**C**) Gross appearance of the excised mass, showing a large, multilobulated mass with a smooth to mildly irregular, glistening dark red to red-brown external surface and multifocal dark purple-red discoloration.

A ventral midline celiotomy was performed. On entry into the abdominal cavity, a large hepatic mass occupying most of the surgical field was immediately visible (Figure 3A). The mass was confirmed intraoperatively to arise from the right lateral liver lobe. Although liver lobectomy had been anticipated, the mass could be separated from the surrounding hepatic parenchyma along a distinct dissection plane. Therefore, local excision of the mass without liver lobectomy was performed using blunt dissection and a vessel-sealing device (LigaSure). The vessel-sealing device was used for tissue division and vascular control. Intraoperative blood loss was not quantitatively measured; however, no active bleeding was observed after removal of the mass. The resection bed was covered with oxidized regenerated cellulose (Surgicel) for adjunctive hemostasis. Figure 3B shows the abdominal cavity after removal of the mass. Grossly, the excised mass was large and multilobulated, with a smooth to mildly irregular, glistening dark red to red-brown external surface and multifocal dark purple-red discoloration (Figure 3C). No clinically significant adhesions or other remarkable intra-abdominal abnormalities were observed. Immediately after mass removal, while the dog continued to receive a 5% dextrose-containing isotonic crystalloid solution at 5 mL/kg/h, the blood glucose concentration was 203 mg/dL. No additional glucose bolus was administered intraoperatively after the preoperative administration of a 20% glucose solution (1 mL/kg, IV). The linea alba and subcutaneous tissues were closed in simple continuous patterns using 3-0 polydioxanone, and the skin was closed with 3-0 nylon in a simple interrupted pattern.

Postoperatively, the 5% dextrose-containing crystalloid solution was continued for 6 h. No hypoglycemia recurred during this period, and no additional glucose bolus was required. The intravenous fluid was subsequently changed to 0.9% sodium chloride, after which no further hypoglycemia was documented.

Postoperatively, the dog maintained a good appetite and activity level, and the blood glucose concentration remained within or near the reference interval without recurrence of clinically significant hypoglycemia. The perioperative changes in selected clinicopathologic variables are summarized in Table 1.

On POD 0, the hematocrit, white blood cell count, and platelet count decreased to 23.7%, 2.38 × 10^3^/μL, and 35 × 10^3^/μL, respectively. The concurrent postoperative decreases in all three cell lines were considered multifactorial, although the exact causes could not be determined. Perioperative blood loss, which was not quantitatively measured, may have contributed to the anemia. The simultaneous leukopenia and thrombocytopenia suggested additional transient perioperative effects, including redistribution and/or consumption of circulating cells; a dilutional contribution from perioperative fluid administration could not be excluded. On POD 1, the hematocrit further decreased to 14.0%. Blood typing identified the dog as DEA 1-positive, and crossmatching with the donor blood showed compatibility. Immediately after the hematocrit of 14.0% was confirmed, 50 mL of whole blood (approximately 13.9 mL/kg) was transfused. An immediate post-transfusion hematocrit was not recorded; the hematocrit measured on the following day was 22.1%. AST increased markedly in the immediate postoperative period (>1000 U/L on postoperative day 1) and then gradually decreased, whereas ALT remained above the upper reference limit throughout the early postoperative period. On postoperative day 4, the D-dimer concentration increased to >10,000 ng/mL. Considering the recent surgery, underlying neoplasia, and marked postoperative increase in D-dimer concentration, clopidogrel was initiated empirically at 2 mg/kg PO every 24 h to reduce the risk of platelet-mediated thrombotic complications. During treatment, serial complete blood cell counts, platelet counts, and D-dimer concentrations were evaluated, and the dog was monitored clinically for signs of hemorrhage or thromboembolism. Clopidogrel was administered for 2 days and was discontinued on postoperative day 5 when the D-dimer concentration showed a decreasing trend. Postoperative hepatoprotective treatment consisted of silymarin (10 mg/kg PO every 12 h), ursodeoxycholic acid (10 mg/kg PO every 12 h), and S-adenosyl-L-methionine (SAMe; 100 mg/tablet, 27.8 mg/kgPO every 24 h). The dog was discharged on postoperative day 5 at the owner’s request. Follow-up testing showed gradual improvement in the postoperative abnormalities. By postoperative day 13, the D-dimer concentration had decreased to 206 ng/mL. On postoperative day 26, blood glucose remained within the reference interval, and the ALT, AST, ALP, and GGT activities were 65, 42, 426, and 11 U/L, respectively. Because the dog remained clinically stable, silymarin, ursodeoxycholic acid, and SAMe were continued for an additional month after postoperative day 26 and were then discontinued.

**Figure 4:**
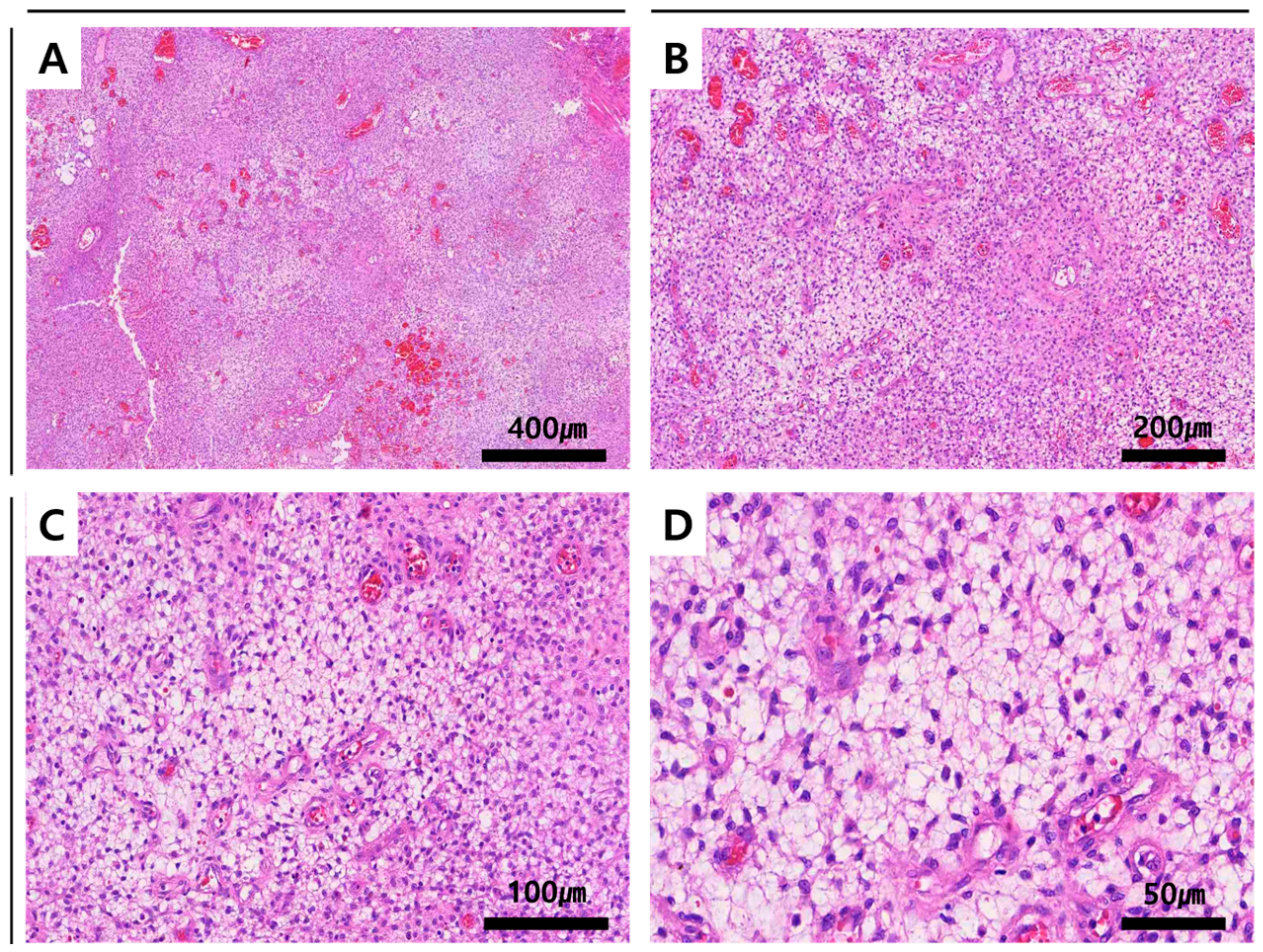
Histopathologic findings of the primary hepatic neoplasm of uncertain histogenesis. (**A**) Low-magnification image showing a poorly demarcated, highly cellular neoplasm with multifocal hemorrhage. (**B**) Neoplastic cells arranged in irregular interlacing bundles and whorls. (**C**) The neoplastic population consisted predominantly of spindle-shaped to ovoid cells with indistinct cell borders and eosinophilic to vacuolated cytoplasm. (**D**) At higher magnification, neoplastic cells exhibited clear to finely vacuolated cytoplasm and centrally located ovoid-to-elongated nuclei. Although cytoplasmic vacuolation prompted consideration of possible adipocytic differentiation, unequivocal lipoblasts were not identified. Hematoxylin and eosin staining. Scale bars = 400 μm (**A**), 200 μm (**B**), 100 μm (**C**), and 50 μm (**D**).

Histopathologic examination of the available material, comprising one cross-section of the submitted hepatic specimen and two sections taken perpendicular to this axis, revealed replacement of the normal hepatic architecture by a poorly demarcated, highly cellular neoplasm with multifocal hemorrhage and necrosis (Figure 4A). The original number of paraffin blocks or cassettes could not be retrospectively confirmed. The neoplastic cells were arranged in interlacing bundles and whorls and ranged from spindle-shaped to ovoid (Figure 4B). Cell borders were indistinct, and the cells contained moderate amounts of fibrillar eosinophilic cytoplasm. Focally, the neoplastic cells exhibited prominent clear to finely vacuolated cytoplasm (Figure 4C,D), prompting consideration of possible adipocytic differentiation; however, the vacuolated cells lacked unequivocal morphological features of lipoblasts. The nuclei were centrally located, ovoid to elongated, and contained vesicular chromatin and one variably prominent nucleolus (Figure 4D). The degree of cellular and nuclear pleomorphism was not formally graded in the available pathology reports. Five mitotic figures were identified in 10 high-power fields at ×400 magnification. Scattered well-differentiated vascular channels were present, but overt vasoformative differentiation was not identified. No intravascular tumor emboli were identified in the examined sections. Because of the limited available sections, local invasion and histologic margin status could not be definitively assessed. Furthermore, because the histogenesis of the neoplasm remained uncertain, a specific histologic subtype and grade were not assigned. The lesion was, therefore, conservatively classified as a primary hepatic neoplasm of uncertain histogenesis.

**Figure 5:**
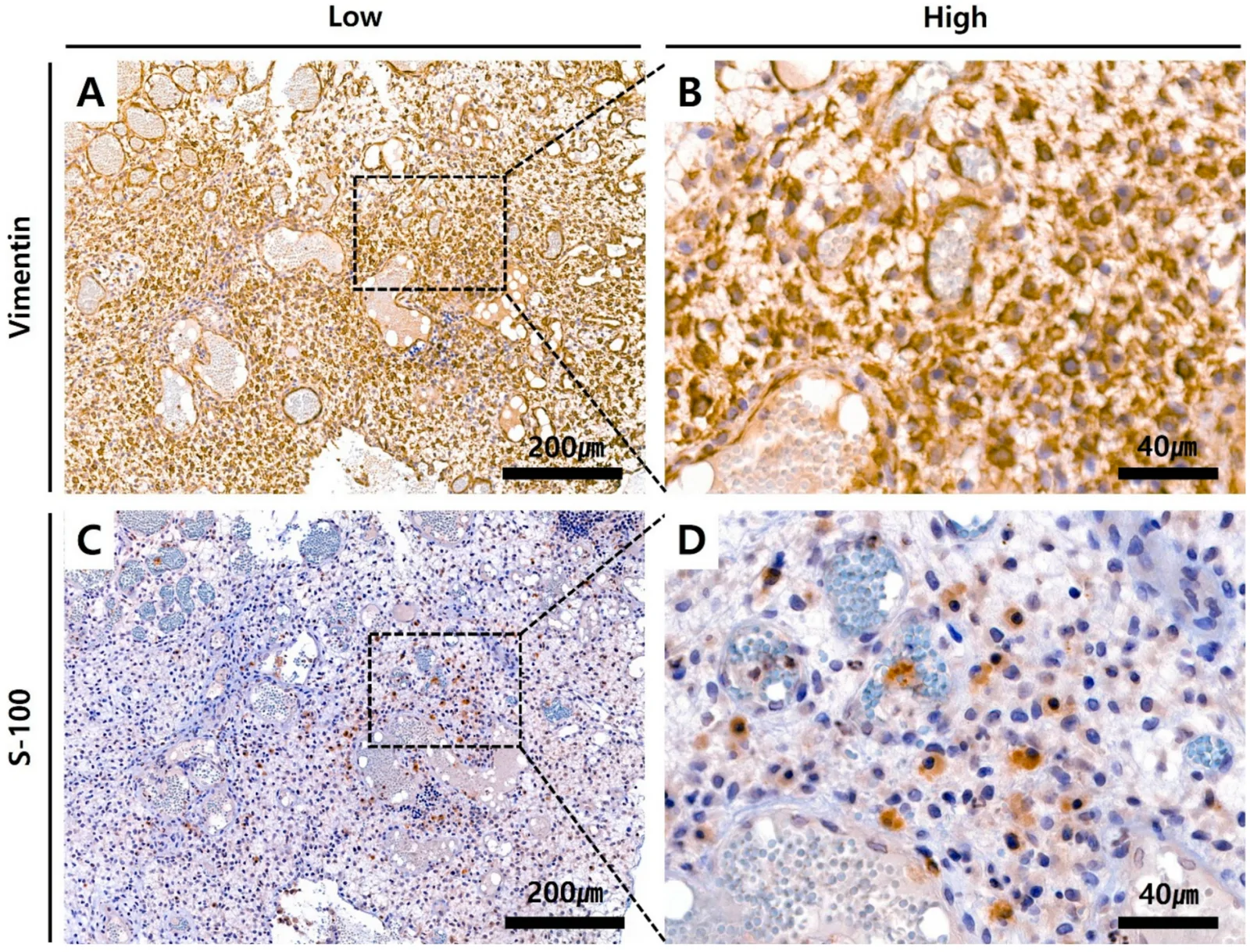
Representative immunohistochemical images of vimentin and S-100. (**A**,**B**) Low- and high-power photomicrographs showing diffuse, strong cytoplasmic immunoreactivity for vimentin in neoplastic cells. Scale bars = 200 μm (**A**) and 40 μm (**B**). (**C**,**D**) Low- and high-power photomicrographs showing weak cytoplasmic immunoreactivity for S-100 in a subset of neoplastic cells. Scale bars = 200 μm (**C**) and 40 μm (**D**).

Immunohistochemical staining was performed at PATH (Pathology for Animal Total Healthcare, Seoul, Republic of Korea), a veterinary diagnostic pathology laboratory. Detailed immunohistochemical methods and antibody-specific information are provided in Supplementary Methods S1 and Table S1. Immunohistochemically, the neoplastic cells showed diffuse, strong cytoplasmic immunoreactivity for vimentin in nearly all neoplastic cells (Figure 5A,B). S-100 immunoreactivity was detected in the cytoplasm of a subset of neoplastic cells, and the staining intensity was weaker than that observed for vimentin (Figure 5C,D).

**Figure 6:**
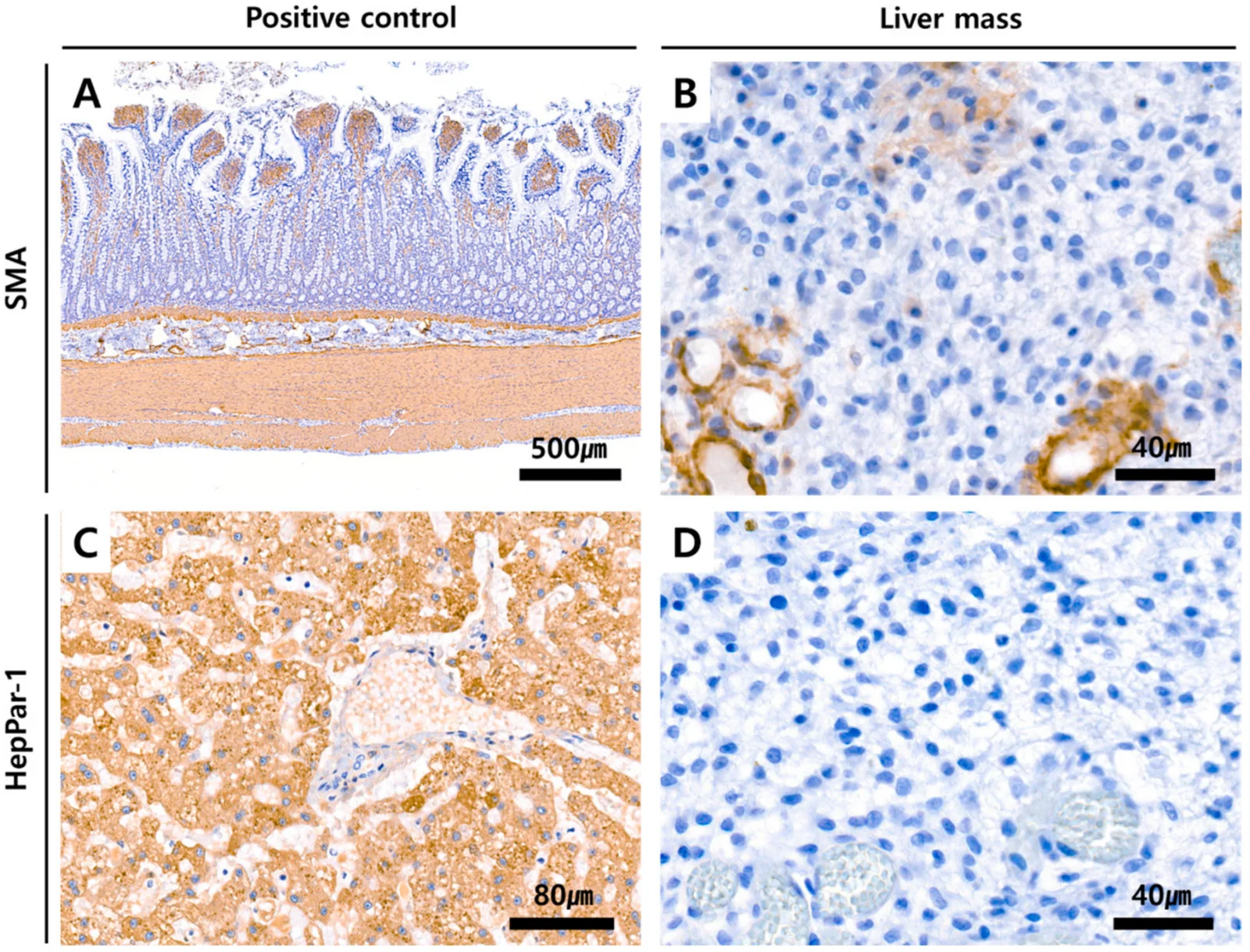
Representative immunohistochemical images of smooth-muscle actin (SMA) and HepPar-1 with corresponding external positive-control tissues. (**A**) External positive control for SMA in normal intestinal tissue, showing the expected immunoreactivity in the smooth-muscle layer. Scale bar = 500 μm. (**B**) No immunolabeling for SMA was observed in the neoplastic cells of the hepatic mass. Scale bar = 40 μm. (**C**) External positive control for HepPar-1 in normal liver, showing the expected hepatocellular immunoreactivity. Scale bar = 80 μm. (**D**) No immunolabeling for HepPar-1 was observed in the neoplastic cells of the hepatic mass. Scale bar = 40 μm. Suitable internal non-neoplastic structures were not available for assessment in the tumor sections; therefore, staining adequacy was assessed using the external positive-control tissues shown in panels (**A**,**C**).

No immunolabeling for smooth muscle actin (SMA) or HepPar-1 was observed in the neoplastic cells (Figure 6). Suitable internal non-neoplastic structures were not available for assessment in the tumor sections; however, the corresponding external positive-control tissues showed the expected immunoreactivity. Diffuse vimentin immunoreactivity supported a mesenchymal phenotype but was not lineage-specific. S-100 immunoreactivity, considered together with the occasional cytoplasmic vacuolation observed histologically, raised the possibility of adipocytic differentiation but was insufficient to establish it. The absence of SMA immunoreactivity made smooth-muscle differentiation less likely. HepPar-1 negativity did not support hepatocellular differentiation but did not definitively exclude hepatocellular carcinoma. As the combined histomorphologic and immunohistochemical findings did not conclusively establish a specific lineage, the lesion was conservatively classified as a primary hepatic neoplasm of uncertain histogenesis.

At 247 days after surgery, the dog remained clinically well. According to the owner, no further episodes of pelvic limb weakness, collapse, or other signs suggestive of hypoglycemia had occurred. The blood glucose concentration remained within the reference interval. Hematologic findings were largely stable, whereas serum biochemical testing revealed increased hepatobiliary enzyme activities, with ALP activity showing the most marked increase at 2031 U/L (Table 1). The cause of the marked elevation in ALP was not definitively determined. Residual biliary sludge and mild diffuse hepatic parenchymal changes were possible contributors; however, no imaging evidence of biliary obstruction was identified. At this follow-up examination, right lateral and ventrodorsal thoracic and abdominal radiographs were obtained, and a complete abdominal ultrasonographic examination was performed. Thoracic radiographs showed no evidence of pulmonary metastasis. Abdominal ultrasonography revealed no evidence of local recurrence or intra-abdominal metastasis. The amount of biliary sludge had decreased compared with that in the previous examination. Mild diffuse changes in the hepatic parenchyma, including increased echogenicity and altered echotexture, were noted, while increased renal cortical echogenicity and chronic cortical changes in both kidneys were similar to those observed previously. No additional antitumor treatment had been administered during the follow-up period.

## 3. Discussion

This case describes recurrent severe hypoglycemia in a dog with a large hepatic neoplasm, followed by the sustained absence of clinically significant hypoglycemia after surgical excision. Although perioperative blood glucose concentrations were influenced by dextrose supplementation, no further clinically significant hypoglycemic episodes were documented after dextrose-containing fluids were discontinued, and clinically significant hypoglycemia was not documented during the subsequent 247-day follow-up period without supplemental glucose or glucocorticoid treatment. This temporal relationship supported an association between the hepatic neoplasm and hypoglycemia. However, neither the precise lineage of the neoplasm nor the specific mechanism underlying the hypoglycemia could be established.

Several potential causes of hypoglycemia were considered during the diagnostic workup [19]. Insulinoma was an important differential diagnosis because it is a common neoplastic cause of hypoglycemia in dogs and is generally supported by the concurrent presence of hypoglycemia and an insulin concentration that is inappropriately within or above the reference interval [19,20]. In this case, the serum insulin concentration measured during documented hypoglycemia was low, and no pancreatic mass was identified on diagnostic imaging. These findings did not completely exclude insulinoma because insulin was measured only once, precluding assessment of temporal variability and reproducibility, and a small or occult pancreatic lesion may not be detected on imaging. Nevertheless, when considered together, the low measured insulin concentration and absence of an identifiable pancreatic mass made insulinoma less likely; however, this interpretation should be made cautiously because formal canine validation and analytical sensitivity data for the insulin assay were not available. Severe hepatic dysfunction was also considered because advanced hepatic disease may cause hypoglycemia [19]. At presentation, hepatobiliary enzyme activities and total bilirubin concentration did not indicate marked hepatocellular injury or cholestasis. Serum albumin concentration and the available preoperative PT and aPTT results were within their respective reference intervals, whereas the blood urea nitrogen concentration was decreased. However, these variables did not comprehensively assess hepatic functional reserve, and serum cholesterol, bile acids, and ammonia were not measured. Therefore, although severe functional hepatic failure was not supported as the primary explanation for the hypoglycemia, impaired hepatic glucose production related to reduced functional hepatic mass could not be completely excluded as a contributing mechanism. Sepsis was also considered because it is a recognized cause of hypoglycemia in dogs. Although leukocytosis and an increased C-reactive protein concentration indicated systemic inflammation, no infectious focus or hemodynamic instability was identified, making sepsis a less likely primary cause [21]. Hypoadrenocorticism, including atypical hypoadrenocorticism, was not supported because the post-ACTH cortisol concentration was 20.8 μg/dL, whereas canine hypoadrenocorticism is confirmed by a post-ACTH cortisol concentration below 2 μg/dL or below the laboratory-specific diagnostic cutoff [18]. Collectively, the presence of a large hepatic mass, a low serum insulin concentration during hypoglycemia, and the prompt and sustained resolution of hypoglycemia after tumor excision supported an association between the hepatic neoplasm and the hypoglycemia, although the specific underlying mechanism could not be determined.

Prednisolone administration was temporally associated with temporary or partial improvement in glycemic control; however, recurrent hypoglycemic episodes continued, and this response was not diagnostically specific. Glucocorticoids can increase blood glucose concentrations through general metabolic effects and have also been used to improve glycemic control in patients with non-islet-cell tumor hypoglycemia [22,23]. Therefore, the partial response to prednisolone did not distinguish tumor-associated hypoglycemia from other potential causes.

Several mechanisms have been proposed for hypoglycemia associated with nonpancreatic tumors, including increased glucose utilization by the tumor, reduction in functional hepatic mass, altered hepatic glucose production, and insulin-like growth factor (IGF)-mediated effects [23,24]. In non-islet-cell tumor hypoglycemia (NICTH), incompletely processed IGF-2 precursors, commonly referred to as high-molecular-weight or big IGF-2, may promote peripheral glucose uptake and suppress hepatic glucose production, resulting in hypoglycemia accompanied by low circulating insulin concentrations [23,24,25]. Most mechanistic understanding of NICTH is derived from human medicine, in which the syndrome has been reported in association with both epithelial and mesenchymal nonpancreatic tumors [24,25,26]. However, the relative contributions of the proposed mechanisms could not be distinguished in the present case. Tumor glucose utilization and hepatic glucose production were not directly assessed. Beta-hydroxybutyrate was not measured. IGF-1 was not measured, and serum IGF-2, the IGF-2:IGF-1 ratio, and high-molecular-weight IGF-2 were not evaluated because validated canine assays for IGF-2-related testing are limited and not routinely available [27]. IGF-2 immunohistochemistry was not performed because it was not available through the diagnostic laboratories involved in this case. Therefore, the condition was interpreted as tumor-associated hypoinsulinemic hypoglycemia with suspected NICTH; however, the contribution of an IGF-mediated mechanism remained unconfirmed.

Preoperative computed tomography was useful for localizing and characterizing the hepatic mass, evaluating its mass effect, assessing for an identifiable pancreatic lesion and pulmonary metastases, and planning surgical treatment. The mass measured approximately 28 HU on precontrast images, and no areas of macroscopic fat attenuation were identified. During the postcontrast phases, the mass showed limited enhancement and remained markedly hypoattenuating relative to the normal hepatic parenchyma. The mean attenuation values of the mass and normal hepatic parenchyma were 23 and 63 HU during the arterial phase, 32 and 87 HU during the portal venous phase, and 48 and 146 HU during the delayed phase, respectively. The mean attenuation of the mass increased by approximately 20 HU between the precontrast and delayed-phase images. Previous studies in dogs have reported that a greater maximal transverse diameter is associated with malignant focal hepatic lesions and that lower delayed-phase absolute enhancement may aid in differentiating malignant from benign hepatic masses [28,29]. Accordingly, the large size and limited delayed-phase enhancement of the mass in the present case raised concern for malignant hepatic neoplasia. However, substantial overlap exists between the CT features of benign and malignant hepatic lesions [30], and these findings were not specific for a particular tumor lineage. Thus, CT was useful for lesion localization, staging, and surgical planning but could not establish the histopathologic classification of the neoplasm or distinguish among the possible epithelial and mesenchymal hepatic neoplasms.

The histopathologic classification of this neoplasm required cautious interpretation. Although the combination of occasional cytoplasmic vacuolation and weak cytoplasmic S-100 immunoreactivity in a subset of neoplastic cells raised suspicion for adipocytic differentiation, neither finding was specific, and unequivocal lipoblasts were not identified. Diffuse vimentin immunoreactivity supported a mesenchymal phenotype but did not establish a specific lineage and could not independently exclude a poorly differentiated carcinoma, because vimentin expression has been reported in canine primary liver carcinomas, including poorly differentiated carcinoma [16]. Clear-cell hepatocellular carcinoma was also considered because clear-cell morphology is a recognized histologic pattern of canine hepatocellular carcinoma [15,31]. However, definitive hepatocellular architectural features were not identified in the available sections, and no HepPar-1 immunolabeling was observed in the neoplastic cells. HepPar-1 is a sensitive but not universally expressed marker of canine hepatocellular carcinoma; therefore, the absence of immunolabeling did not support hepatocellular differentiation but could not definitively exclude hepatocellular carcinoma [17]. The absence of SMA immunoreactivity made smooth-muscle differentiation less likely [32]. Overall, the limited immunohistochemical panel could not exclude other poorly differentiated epithelial neoplasms or other mesenchymal neoplasms. Consequently, the combined histomorphologic and immunohistochemical findings were insufficient to establish liposarcoma, clear-cell hepatocellular carcinoma, or another specific tumor lineage, and the lesion was conservatively classified as a primary hepatic neoplasm of uncertain histogenesis.

From a clinical perspective, the unresolved tumor lineage indicates that, in a dog with recurrent severe hypoglycemia and a large hepatic mass, the differential diagnosis should extend beyond insulinoma and should not presume a specific hepatic tumor type. Both epithelial and mesenchymal hepatic neoplasms should remain under consideration when other common causes of hypoglycemia are not supported; in this case, these included clear-cell hepatocellular carcinoma and a mesenchymal neoplasm with possible adipocytic differentiation. More definitive classification may require additional representative tissue, integration of histomorphology with a broader immunohistochemical panel, and, where available, molecular or other lineage-specific testing.

Serial blood glucose monitoring documented recurrent severe hypoglycemia and the repeated need for dextrose supplementation before surgery. Dextrose-containing intravenous fluids were administered intraoperatively and maintained for 6 h postoperatively; therefore, early perioperative blood glucose measurements could not be interpreted independently of exogenous glucose administration. However, no recurrent clinically significant hypoglycemia was documented after the dextrose-containing fluids were discontinued, and no clinically significant hypoglycemia was subsequently documented without supplemental glucose or glucocorticoid treatment throughout the 247-day follow-up period. Published canine data on the resolution of hepatic tumor-associated hypoglycemia following surgical resection are limited to a small case series and individual case reports, with most reported hepatic cases involving hepatocellular neoplasms [1,11,12,13,14]. Detailed longitudinal reports integrating diagnostic imaging, medical stabilization, perioperative glucose management, surgical treatment, and postoperative follow-up also remain limited. By documenting these components in chronological sequence, the present case provides clinically useful information for the evaluation and management of dogs with recurrent severe hypoglycemia and a resectable hepatic mass. Furthermore, it provides additional circumstantial evidence that the hepatic neoplasm contributed to the hypoglycemia, despite the absence of direct confirmation of the underlying mechanism. Additionally, because the enlarged hepatic lymph node identified on CT was not sampled, regional nodal metastasis could not be excluded, and regional oncologic staging remained incomplete. At the final examination, no imaging evidence of local recurrence or distant metastasis was identified. This clinical course supports the potential therapeutic value of surgical excision in dogs with recurrent severe hypoglycemia and a resectable hepatic mass, even when the precise tumor lineage and mechanism of hypoglycemia cannot be established. Additional cases with longer follow-up and more comprehensive endocrine and pathologic evaluation are needed to clarify the biological behavior of hepatic neoplasms associated with hypoglycemia and the mechanisms underlying tumor-associated hypoglycemia.

## 4. Conclusions

This case highlights that hepatic neoplasia should be considered as a potential contributor to recurrent severe hypoglycemia when a large hepatic mass is identified, particularly when insulinoma is considered less likely and other common causes of hypoglycemia are not supported. The absence of recurrent clinically significant hypoglycemia for 247 days after surgical excision supports an association between the hepatic neoplasm and hypoglycemia. However, the precise tumor lineage and the specific mechanism of hypoglycemia, including whether an IGF-mediated process was involved, remained unconfirmed.

## Figures and Tables

**Table 1:** Perioperative changes in selected clinicopathologic variables before and after hepatic mass resection.

Parameter	Reference Interval	Pre-Op	POD 0	POD 1	POD 2	POD 3	POD 4	POD 5	POD 13	POD 26	POD 247
HCT (%)	37.3–61.7	39.7	23.7	14.0	22.1	24.8	28.5	34.8	34.8	—	37.2
WBC (×10^3^/μL)	5.05–16.76	12.43	2.38	15.44	14.45	16.87	17.31	14.11	9.72	—	16.76
PLT (×10^3^/μL)	148–484	287	35	77	42	94	201	438	697	—	505
Glucose (mg/dL)	75–128	39	90–203	88–131	65–233	88–124	75–100	78–100	88	110	108
ALT (U/L)	17–78	188	—	>1000	>1000	>1000	>1000	>1000	398	65	123
AST (U/L)	17–44	29	—	>1000	563	—	71	58	38	42	54
ALP (U/L)	47–254	302	—	412	438	—	—	351	708	426	2031
GGT (U/L)	5–14	21	—	8	12	—	—	15	15	11	18
CRP (mg/L)	0–10	5.18	—	107.88	27.85	15.71	—	18.19	<5	—	—
D-dimer (ng/mL)	0–250	198	—	6351.82	3874.76	4355.59	>10,000	9182.97	206	—	—

Abbreviations: ALP, alkaline phosphatase; ALT, alanine aminotransferase; AST, aspartate aminotransferase; CRP, C-reactive protein; D-dimer, cross-linked fibrin degradation product; GGT, gamma-glutamyl transferase; HCT, hematocrit; PLT, platelet count; POD, postoperative day; Pre-op, preoperative; WBC, white blood cell count. Pre-op values were obtained on the day of surgery before anesthetic induction; the preoperative blood glucose concentration represents the final measurement obtained immediately before anesthetic induction. POD 0 indicates the day of surgery. Except for blood glucose, POD 0 values were obtained after completion of surgery. Blood glucose values presented as ranges represent the minimum and maximum concentrations obtained from serial measurements during the corresponding period. The POD 0 blood glucose range includes measurements obtained intraoperatively and during the remainder of the day of surgery. —, not measured.

## Data Availability

The data presented in this study are available from the corresponding author upon reasonable request. Public availability of the data is restricted because they contain clinical information from a client-owned animal.

## Supplementary Materials

The following supporting information can be downloaded at: https://www.mdpi.com/article/10.3390/vetsci13080822/s1, Supplementary Methods S1: Immunohistochemistry; Table S1: Primary antibodies used for immunohistochemical evaluation of the hepatic mass.

## Abbreviations

The following abbreviations are used in this manuscript:

ACTH	adrenocorticotropic hormone
ALP	alkaline phosphatase
ALT	alanine aminotransferase
AST	aspartate aminotransferase
CBC	complete blood count
cPL	canine pancreas-specific lipase
CRP	C-reactive protein
CT	computed tomography
GGT	gamma-glutamyl transferase
HCT	hematocrit
HepPar-1	hepatocyte paraffin 1
HU	Hounsfield unit
IGF	insulin-like growth factor
PLT	platelet count
POD	postoperative day
RI	reference interval
SAMe	S-adenosyl-L-methionine
SMA	smooth muscle actin
T4	thyroxine
WBC	white blood cell count
