# Supplementary material for: Resolution of Recurrent Severe Hypoglycemia After Surgical Excision of a Primary Hepatic Neoplasm of Uncertain Histogenesis in a Dog: A Case Report
Source: Vet Sci. 2026 Aug 18;13(8):822. doi: 10.3390/vetsci13080822 (PMC13517161; doi:10.3390/vetsci13080822)
Supplement: Supplementary file 1 [file vetsci-13-00822-s001.zip › vetsci-4468683-supplementary.pdf]

Table S1. Primary antibodies used for immunohistochemical staining of a hepatic mass.

Antibody	Host	Manufacturer and catalog no.	Clone/ID	Clonality	Dilution	Antigen retrieval	Positive control
Vimentin	Mouse	Dako M0725	V9	mAb	1:200	Citrate	Intestine
S-100	Rabbit	Dako IR504	N/A	pAb	1:300	Tris-EDTA	Melanoma
SMA	Mouse	Dako M0851	1A4	mAb	1:200	Tris-EDTA	Intestine
HepPar-1	Mouse	BioSB PI5629	OCH1E5	mAb	1:500	Citrate	Liver

SMA, smooth muscle actin; pAb, polyclonal antibody; mAb, monoclonal antibody.

<Methods and materials>

Immunohistochemistry (IHC)

IHC staining was carried out using 4-µm sections of formalin-fixed, paraffin-embedded tissue specimens. The tissue was fixed in 10% neutral-buffered formalin; however, the exact fixation duration could not be retrospectively confirmed. Sections were deparaffinized with xylene and then rehydrated through a series of graded ethanol. Heat-induced antigen retrieval was performed in either citrate buffer at pH 6.0 or Tris-EDTA buffer at pH 9.0, depending on the conditions recommended by the antibody manufacturer. Endogenous peroxidase activity was subsequently blocked by treatment with 3% hydrogen peroxide. The tissue sections were incubated for 24 hours at 4 °C with primary antibodies directed against vimentin, S-100, SMA, and Hep-Par-1. All antibodies used in this study had been validated for diagnostic immunohistochemical application. Details regarding antibody clones, dilutions, and manufacturers are provided in Table S1. Appropriate horseradish peroxidase (HRP)-conjugated secondary antibodies (Horse anti-mouse IgG [H+L], Peroxidase, Vector laboratories; goat anti-rabbit IgG (H+L), Peroxidase, Vector Laboratories) corresponding to the host species of each primary antibody were applied for 1 hour at room temperature. Immunoreactivity was subsequently visualized using 3,3′-diaminobenzidine

(DAB; ImmPACT DAB EqV substrate kit, Peroxidase, Vector Laboratories) as the chromogen substrate. The slides were then counterstained with hematoxylin. Appropriate positive and negative controls were included in each staining procedure. For the negative controls, the primary antibodies were omitted and replaced with antibody diluent.

The stained slides were evaluated by light microscopy. Immunoreactivity was assessed according to the distribution and intensity of positive signals.
